# Lupus and Tuberculosis

**Published:** 1891-01-24

**Authors:** 


### The Doctor's Armchair.

### LUPUS AND TUBERCULOSIS.

Mr. Jonathan Hutchinson, F.R.S. delivered a lecture at the Examination Hall, Savoy Street, last week, to the members of the medical profession, on "Lupus in general, with special reference to its connection with tuberculosis." The claim of Dr. Koch to have discovered a remedy for tubercular phthisis rests its proof largely on the assumption that lupus is a form of tubercle; that in fact it is as truly a tubercular disease as is phthisis of the lungs. Medical readers know that it is only within the last few years that lupus has been described as a tubercular affection. A properly scientific discussion of Koch's position and claims requires the settlement of this one preliminary question: Is lupus, or is it not, a truly tubercular disease with a truly tubercular origin? For if it be open to doubt whether lupus be truly tubercular, then clearly the fact that a particular line of treatment favourably affects lupus cannot be held to be in any sense an irrefragable proof that the same treatment will cure or favourably affect tubercular phthisis.

Medical men, it appears to us, have got themselves into a good deal of confusion during the last few years; a confusion which is not to their honour. The pathological laboratory has claimed the position of an oracle; and all ranks of men in the profession have listened to it with a much profounder and more superstitious veneration than did either Greeks or Romans in earlier times to their oracles and auguries on the eve of a critical battle. We have nothing but respect for men who work diligently and honestly in laboratories and who give their conclusions to the world. Even if those conclusions be sometimes wrong we still continue to respect the workers for their honest efforts after truth. But the world is large; the human organism is exceedingly complex; its history strikes deep into the roots of a remote past; its environment includes influences of manifold variety and potency. A calm and judicial survey of man as he is, as he has been, as he is environed, will not permit us to accept conclusions of first-rate magnitude and importance on the sole authority of a few individuals, most of them comparatively young, who have worked for ten or a dozen years in pathological laboratories. The tests of time and of world-wide experience are indispensable.

Mr. Hutchinson's lectures, coming at the present time, are peculiarly opportune. If any man has earned the right to speak as an oracle, Mr. Hutchinson has. But he does not speak as an oracle. All reasonable people who want to get at solid truth will do well to read his lectures, and to think over his positive statements of fact, and his judicial expressions of opinion, with earnest intelligence. These are some of Mr. Hutchinson's claims to be heard: he has been practising surgery for forty years; and we venture to say that during all those forty years not a single hour of his working time has been spent unscientifically. Mr. Hutchinson could not be unscientific if he would. He is as much at home in the laboratory as in the consulting room and at the bedside. Whatever the pathologists and the bacteriologists know from their laboratory experiments about the nature and origin of lupus Mr. Hutchinson knows; whilst he knows a hundred things about its clinical history, its manifestations in an infinite variety of cases, its practical treatment, its effects upon the system, and its affinities with other diseases, which the mere bacteriologists neither do know, nor can know, anything at all about. In listening to Mr. Hutchinson, we are listening to a Cæsar, to a Napoleon the First, to a Wellington, to a Moltke. In listening to a mere bacteriologist we are listening to a teacher at a military academy. Between these two there is all the difference in the world. Mr. Hutchinson is truly scientific, because he knows both the science and the practice: the pure bacteriologists are only partially scientific, because they only know so much of science as they have been able to acquire without practice.

Coming then to the gist and point of this article: Is Mr. Hutchinson convinced that lupus is truly a tubercular disease of the skin? He is not. On the contrary his convictions and his judgment lie all the other way. What he does believe about lupus is that it is a "specialised form of chronic inflammation" of the skin or other part "rather than a disease of bacillary origin." Now, if tubercle be, really and truly, a disease of bacillary origin, whilst lupus is not, then it is clear that the two affections cannot be identical. But Mr. Hutchinson points out a fact, more or less familiar to every medical man, which has the most important bearing on the subject. It is this: that, according to his experience, lupus, at any rate common lupus, seldom, or practically never, infects the lungs or other internal organs of a person who is the subject of it: in other words, no patient who is afflicted with common lupus ever becomes consumptive. What is more, common lupus of the skin does not tend to infect even the nearest lymphatic glands. Now this is a fact of the utmost significance; but it is exactly the kind of fact which the mere laboratory pathologist tends to overlook. He stands by his microscope; and by his microscope he will live or die; and he eventually will die, scientifically, if his microscope be the only rushlight with which he gropes his way about the world of medical and pathological facts.

For let any medical man consider the nature and history of known infectious diseases! Take, for example, the case of cancer which is an infectious disease, but yet, not half so rapidly and powerfully infectious as tubercle. What is the common history of a case of cancer of the breast? The disease begins in a group of mammary lobules; in a few weeks or months it infects the neighbouring lobules; in a few more weeks or months the axillary glands are affected; a little later the intra-thoracic glands fall victims; in no long time the spleen or the liver or both may follow, and other abdominal glands in their turn. In short, if the patient live long enough there may hardly be a gland in the body which remains unaffected. A much more rapid and striking history than this is the case of acute miliary tuberculosis. From a few infective centres in one of the lungs the poison may be carried with lightning-like rapidity to every vital organ of the body; and a person who was but yesterday well and strong may be dead in three months.

If then lupus be a truly tubercular disease, is it not passing strange that so experienced and scientific an observer as Mr. Hutchinson should doubt its bacillary origin; and is it not ten times stranger still that lupus, of every form of tubercle, should have practically no power of infecting any other part of the body but the skin in which it commences and lives? The questions raised by Mr. Hutchinson are of immediately practical importance. A revolution in medical theory and practice is impending. If that revolution should come, and if it should land us in regions of deeper and wider knowledge and of more definite and successful treatment, every medical man will welcome it as he would welcome prosperity and happiness for himself. But there is always the possibility that a revolution may land us in profounder darkness, and in methods less efficient and successful than we now practice. The only way to insure that a revolution shall accomplish good and not harm, or at any rate more good than harm, is to make certain that the impulses which move it are the offspring of proved realities, and that every turn of the wheel is in the direction of more light and truth. It is by hearing both sides, by weighing all facts, and by giving prolonged, and if need be, painful consideration to every fact and reason, that truth is arrived at. He who thinks that scientific advancement is accomplished by kangaroo-leaps or the gyrations of a flying machine is but at the beginning of his alphabet.

